# Tomographic reconstruction with a generative adversarial network

**DOI:** 10.1107/S1600577520000831

**Published:** 2020-02-18

**Authors:** Xiaogang Yang, Maik Kahnt, Dennis Brückner, Andreas Schropp, Yakub Fam, Johannes Becher, Jan-Dierk Grunwaldt, Thomas L. Sheppard, Christian G. Schroer

**Affiliations:** a FS-PETRA, Deutsches Elektronen-Synchrotron DESY, Notkestraße 85, D-22607 Hamburg, Germany; b MAX IV Laboratory, Lund University, 22100 Lund, Sweden; c Department Physik, Universität Hamburg, Luruper Chaussee 149, D-22761 Hamburg, Germany; d Faculty of Chemistry and Biochemistry, Ruhr-University Bochum, Universitätsstraße 150, 44801 Bochum, Germany; e Institute for Chemical Technology and Polymer Chemistry, Karlsruhe Institute of Technology, Engesserstraße 20, 76131 Karlsruhe, Germany; f Institute of Catalysis Research and Technology, Karlsruhe Institute of Technology, Hermann-von-Helmholtz Platz 1, 76344 Eggenstein-Leopoldshafen, Germany

**Keywords:** missing-wedge tomography, reconstruction algorithms, generative adversarial network (GAN), ptychography

## Abstract

This paper presents a deep learning algorithm for tomographic reconstruction (GANrec). The algorithm uses a generative adversarial network (GAN) to solve the inverse of the Radon transform directly. It works for independent sinograms without additional training steps. The GAN has been developed to fit the input sinogram with the model sinogram generated from the predicted reconstruction. Good quality reconstructions can be obtained during the minimization of the fitting errors. The reconstruction is a self-training procedure based on the physics model, instead of on training data. The algorithm showed significant improvements in the reconstruction accuracy, especially for missing-wedge tomography acquired at less than 180° rotational range. It was also validated by reconstructing a missing-wedge X-ray ptychographic tomography (PXCT) data set of a macroporous zeolite particle, for which only 51 projections over 70° could be collected. The GANrec recovered the 3D pore structure with reasonable quality for further analysis. This reconstruction concept can work universally for most of the ill-posed inverse problems if the forward model is well defined, such as phase retrieval of in-line phase-contrast imaging.

## Abstract 2

A generative adversarial network (GAN) is used to reconstruct the missing-wedge tomographic data of an *in situ* ptychographic measurement.

## 1. Introduction   

Tomographic imaging is becoming a common tool for many different X-ray imaging techniques at synchrotron light sources, such as transmission X-ray microscopy (TXM), X-ray fluorescence (XRF) imaging and X-ray ptychography (Dierolf *et al.*, 2010 ▸; Mino *et al.*, 2018 ▸). Tomographic reconstruction is a key procedure to assign the scanning signal from different angles to the internal structure of the objects. The mathematical theory of tomographic reconstruction has been well developed for more than half a century (Landis & Keane, 2010 ▸). However, the development of reconstruction algorithms is still a challenge due to possible imperfections in the measurement data. This is particularly the case for synchrotron radiation applications, as the instrumental setup and data quality vary greatly between different beamlines. Pre-processing of the experimental data is always necessary and advanced development of reconstruction algorithms is also essential for some extreme cases, such as missing-wedge tomography. For example, X-ray microscopy is often applied in two dimensions to measure functional materials, *e.g.* catalysts under specific gas and temperature conditions (de Smit *et al.*, 2008 ▸; Grunwaldt & Schroer, 2010 ▸). Such experiments can provide a great deal of information on sample composition, structure and stability, but require the use of dedicated sample environments (*in situ* cells). However, extending such studies to 3D tomography inevitably blocks sample visibility across a 180° rotation range, due to eclipsing of the incoming X-rays by the *in situ* cell body, leading to missing-wedge artefacts. This issue has not been adequately resolved, and *in situ* nano-tomography studies therefore remain challenging.

The reconstruction of missing-wedge tomography suffers from strong artefacts that generate erroneous structures of the object. These artefacts can be reduced from pre-processing of the sinogram (Kudo & Saito, 1991 ▸; Huang *et al.*, 2017 ▸), during reconstruction (Kupsch *et al.*, 2016 ▸), or post-processing of the reconstruction. Pre-processing often involves filling in the missing projections of the sinogram. However, this comes with the risk of producing even more additional artefacts because there are not enough constraints on the missing data. On the other hand, post-processing can be used to correct the strip artefacts of the reconstruction but it cannot recover the missing structure of the object.

A safe and efficient alternative to these methods is to recover the missing-wedge information directly during reconstruction. According to the analytical reconstruction theories, the missing wedge leads to missing information in Fourier space. A reconstruction algorithm cannot recover this information with direct inversion, but reconstruction algorithms based on global optimization make it possible. When we forward project the reconstructions with missing-angle artefacts, there will also be artefacts and errors in the projected sinograms. The reconstruction artefacts can be corrected during the process of minimizing the errors between the projected and input sinograms. Traditional optimization algorithms are ineffective for this case because the constraints on the missing angles are much weaker than for the scanned angles. The iterations accumulate the errors from this unbalanced constraint, causing a local minimum in the convergence of the reconstruction process. Thus, possible routes to mitigate these errors using traditional optimization algorithms are challenging.

Deep neural networks (DNNs) have shown potential improvements for X-ray image processing in recent years (Yang *et al.*, 2017 ▸, 2018 ▸), and have also been developed for tomographic reconstructions. The image-to-image models of convolutional neural networks (CNNs) were previously used as a post-processing method for limited-data tomography (Pelt *et al.*, 2018 ▸; Pelt & Batenburg, 2013 ▸; Hammernik *et al.*, 2017 ▸; Zhang *et al.*, 2016 ▸). These are typical supervised learning routines, for which training data are needed. The CNNs were also coupled with filtered back projection (FBP), resulting in an iterative reconstruction algorithm (Jin *et al.*, 2017 ▸). Recently, a direct tomographic reconstruction using DNN has been developed (Zhu *et al.*, 2018 ▸). These reports all showed improvements for missing-wedge reconstruction using the DNN.

A generative adversarial network (GAN) couples two different networks, a generator and a discriminator, to produce the image as the training target (Goodfellow *et al.*, 2014 ▸). The generator is a network that translates the random initialized noise signal or specific input signal to be the candidate image. The discriminator is a CNN classifier that evaluates how close the candidate image is to the target image. The discriminator can evaluate the image quality with better accuracy than typical cost function approaches because it is a feature-based analysis. It can fit the reconstruction to the target image with good accuracy in the standards of image quality, which is not possible to evaluate by any single criterion. GANs are popular for generating target images with expected shapes and styles using supervised learning routines (Isola *et al.*, 2017 ▸). They were previously applied to sinogram completion for limited-angle tomography (Yoo *et al.*, 2019 ▸; Li *et al.*, 2019 ▸). However, these methods follow the supervised learning workflow and therefore it is difficult to define a sufficient training data set, given that synchrotron tomography studies are performed on a highly diverse range of measurement objects and have strong variations in data quality.

In this paper, we present a tomographic reconstruction technique based on a GAN (GANrec). It can directly reconstruct the sinogram to be the final reconstruction, without the additional training procedures required by other deep learning approaches. The algorithm first transforms the sinogram to a candidate reconstruction with the generator of the GAN. We then use the Radon transform (Barrett, 1984 ▸) to get a model sinogram from this candidate reconstruction. The model sinogram is compared with the input sinogram by the discriminator of the GAN. A good-quality reconstruction can be obtained when the model sinogram is very close to the input sinogram after several iterations. We evaluated the GANrec with missing-wedge tomographic projections of a 3D phantom object, showing better accuracy of reconstruction than traditional algorithms. The GANrec was able to reconstruct the projections of up to 60° missing angles without any visible artefacts and noise, for which the traditional algorithms failed to get a reasonable result. We further validated the GANrec with a real X-ray ptychographic tomography (PXCT) measurement of a zeolite particle deposited on a MEMS chip (Weissenberger *et al.*, 2019 ▸), developed as part of a dedicated sample environment for *in situ* ptychography studies (Fam *et al.*, 2019 ▸). Only 51 projections in 70° were scanned due to the geometric limitations of the setup and time constraints. The GANrec reconstructed the image with sufficient quality for quantitative analysis. The effectiveness of the GANrec for mitigating missing-wedge artefacts synergizes with new applications of PXCT in dedicated sample environments (*in situ* cells) where the full angular range of 180° is not accessible.

## 2. Method   

**Figure 1:**
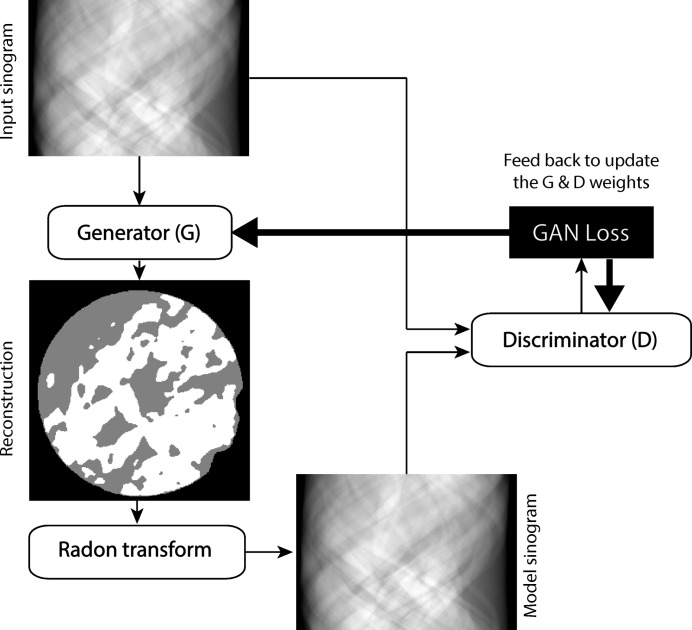
The flowchart of the GANrec algorithm. The input of the GANrec is the sinogram to be reconstructed. The sinogram is transformed into a candidate reconstruction by the generator of the GAN algorithm. The candidate reconstruction is projected to a model sinogram by the Radon transform. The model sinogram is compared with the input sinogram by the discriminator of the GAN. A GAN loss is obtained from this comparison. The weights of the generator and discriminator of the GAN are updated by optimizing the GAN loss.

We developed the GANrec by integrating the Radon transform in the conditional GAN (Isola *et al.*, 2017 ▸). We designed a special generator (*G*) to transform the sinogram to a reconstruction, which is not possible with the typical U-net (Ronneberger *et al.*, 2015 ▸). We used a typical CNN classifier as the discriminator (*D*). The generator transforms the input sinogram as a candidate reconstruction. A model sinogram is then generated from the candidate reconstruction by the Radon transform. The discriminator compares the model sinogram with the input sinogram and provides feedback to the generator as to whether the reconstruction is accurate or not. By repeating this for many iterations, the weights of the generator are fitted to transform the input sinogram to match the correct reconstruction (Fig. 1 ▸).

### 2.1. Objective   

The overall objective of the GANrec can be expressed as
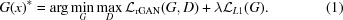
Here, *G*(*x*)* is the candidate reconstruction generated from the input sinogram *x*. The reconstruction process, which can also be considered as the training process, is done while *G* tries to minimize the objective against an adversarial *D* that tries to maximize it.


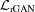
 is the loss of the GANrec. We use the sigmoid cross entropy loss function (Isola *et al.*, 2017 ▸) to calculate it:
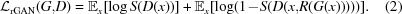
Here 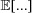, *S*(…) and *R*(…) denote the cross entropy operator, the sigmoid operator and the Radon transform, respectively.

In addition to the GANrec loss, we also use a penalized *L*_1_ loss 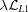 to help the convergence,
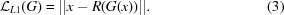



### 2.2. Architecture of the networks   

**Figure 2:**
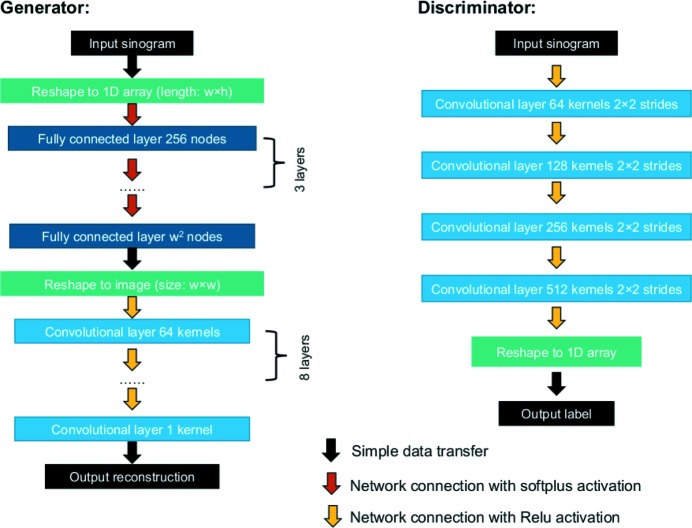
The network architectures of the generator and the discriminator. The generator is formed with four fully connected layers and nine convolutional layers. The discriminator is formed with four convolutional layers. The Softplus activation function (Nwankpa *et al.*, 2018 ▸) is used to connect the fully connected layers. The ReLU activation function (rectified linear unit; Xu *et al.*, 2015 ▸) is used to connect the convolutional layers.

We use a mixing architecture for the generator (*G*) (Fig. 2 ▸). It starts with four fully connected layers, followed by nine convolutional layers. The input sinogram is first transformed into a 1D array. The fully connected layers convert this 1D array to the data domain as the target reconstruction. The converted 1D array is then transformed back to a 2D image. The convolutional layers process this 2D image to find the best-fitting features of the target reconstruction and generate the final image as the candidate reconstruction.

The sinogram-to-reconstruction transformation can be done directly with a single fully connected layer (Paschalis *et al.*, 2004 ▸). The accuracy can be improved by increasing the numbers of layers and nodes, but these numbers are limited by the available computational power. In addition, the fully connected layer connects each pixel to each node of the layer, which requires a huge number of weights. Noise and artefacts can easily be generated due to the difficulty of fitting these randomly initialized weights. The convolutional layer requires a much smaller number of weights than the fully connected layer for the image process. We use the convolutional layers to refine the reconstruction further to give the final candidate reconstruction.

The typical generators for image-to-image translation models of GANs use the encoder–decoder network (Pathak *et al.*, 2016 ▸; Wang & Gupta, 2016 ▸; Johnson *et al.*, 2016 ▸; Yoo *et al.*, 2016 ▸; Zhao *et al.*, 2016 ▸), or its improved version ‘U-Net’ (Ronneberger *et al.*, 2015 ▸; Isola *et al.*, 2017 ▸). These do not work for the sinogram-to-reconstruction transformation since the input and output images are not in the same domain.

We designed the discriminator including four convolutional layers with strides of factor two. The input images of the discriminator are the model sinogram and the input sinogram. The architecture of the discriminator is very similar to the typical CNN classifier. It extracts feature pools of the input image in four scales while downsampling the image with the strides of the convolutional layers. The difference from the typical classifier is that we do not use a fully connected layer at the end. The output of the network is the 1D array of the feature pool from the last convolutional layer.

### 2.3. Normalization and optimization   

The network changes the input data to a different range. The input and output for both the generator and discriminator all need to be normalized. We did the normalization in two steps, *I*_1_ = 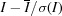 and *I*_2_ = [*I*_1_ − min(*I*_1_)]/[max(*I*_1_) − min(*I*_1_)]. This procedure ensures that the input and output of the networks are always in a comparable range for the convergence of the optimization process. These normalization steps were applied to the input sinogram, the reconstruction from the generator, and the model sinogram, as shown in Fig. 1 ▸.

We also used a layer normalization for each layer of the network (Lei Ba *et al.*, 2016 ▸), which helped to speed up the convergence and improve the accuracy of the final results in our tests.

We used the standard optimization routine of the GAN for the *G* and *D* (Goodfellow *et al.*, 2014 ▸) for the objective function [equation (1)]. In a different approach from the standard routine, we used the Adam optimizer for both *G* loss and *D* loss (Kingma & Ba, 2015 ▸), which showed the best convergence and stability for our application.

## 3. Results   

### 3.1. Evaluation of the GANrec with synthetic data   

**Figure 3:**
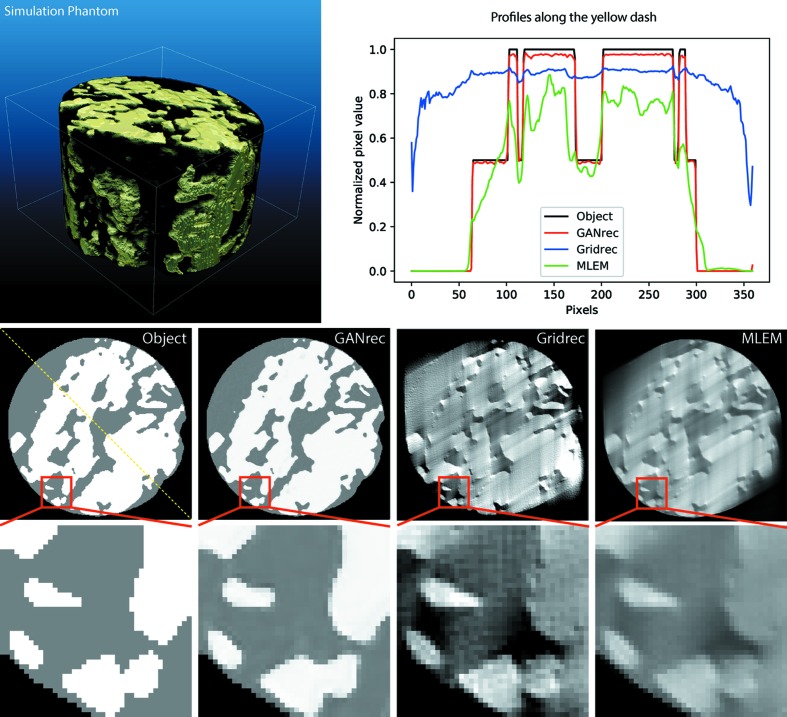
The 3D phantom for the evaluation (top left) and three reconstructions compared with the object of the ground truth (middle row). The plot in the top right shows the profiles along the yellow dashed line. The 3D phantom is extracted from a real micro-CT measurement of a shale sample. Its size is 160 × 256 × 256 pixels. We simulated the sinogram from 120 projections within a limited angular range of 0–120°, *i.e.* 1° steps, and reconstructed with the GANrec, Gridrec and MLEM algorithms, respectively. The pixel value range of the images is scaled to 0–1 for comparison. The brightness and contrast of the Gridrec and MLEM results are optimized to show the best structure of the object. The bottom row shows enlargements of the areas outlined in red in the respective images in the middle row.

We evaluated the performance of the GANrec algorithm with a simulation phantom, extracted from the 3D structure of a shale sample (Fig. 3 ▸, top left). The original data were collected in TomoBank (Carlo *et al.*, 2018 ▸) and were measured with microCT on the TOMCAT beamline of the Swiss Light Source (Paul Scherrer Institut, Villigen, Switzerland). We first used *Tomopy* (Gürsoy *et al.*, 2014 ▸) to make the reconstruction for the original data. The simulation phantom was then extracted by doing segmentation for the original reconstruction with a deep learning method (Shashank Kaira *et al.*, 2018 ▸). We used this simulation phantom to generate seven groups of tomographic projections with scanning angles of 0–180°, 0–170°,…, 0–120°, with one projection per degree. We reconstructed these tomographic projections with the GANrec algorithm and compared the result with those of two other algorithms: the Fourier grid reconstruction algorithm (Gridrec) of *Tomopy* (Gürsoy *et al.*, 2014 ▸) and the maximum-likelihood expectation maximization algorithm (MLEM) of *Astra* (Pelt *et al.*, 2016 ▸).

**Figure 4:**
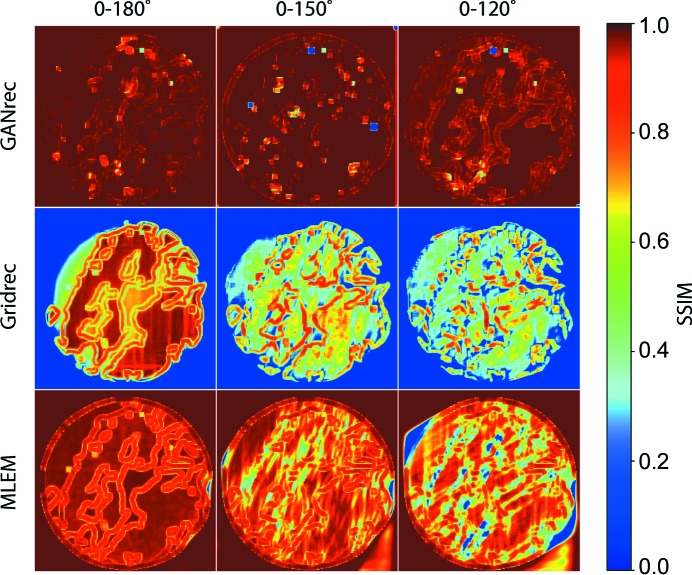
The structure similarity index map (SSIM) of the reconstructions compared with ground truth. A higher SSIM value (red) indicates better reconstruction accuracy.

The middle row of Fig. 3 ▸ shows the sample slices of the reconstructions for the 0–120° projections. The GANrec algorithm reconstructed the object with high accuracy. The traditional algorithms (Gridrec and MLEM) show very strong noise and the overall structure deformed as expected. We plotted the structural similarity index map (SSIM) of the reconstructions compared with the objective image (Fig. 4 ▸). The reconstructions of the GANrec algorithm showed a very high SSIM value (>0.98) even with a 30° or 60° missing wedge, with no significant quality loss when missing up to 60° projections. Only a few tiny spots had a low SSIM value (the blue spots in Fig. 4 ▸ first row). These spots are randomly distributed and have a negligible influence on the 3D analysis. The traditional algorithms, Gridrec and MLEM, showed the typical missing and deformed structures expected from a missing-wedge reconstruction.

**Figure 5:**
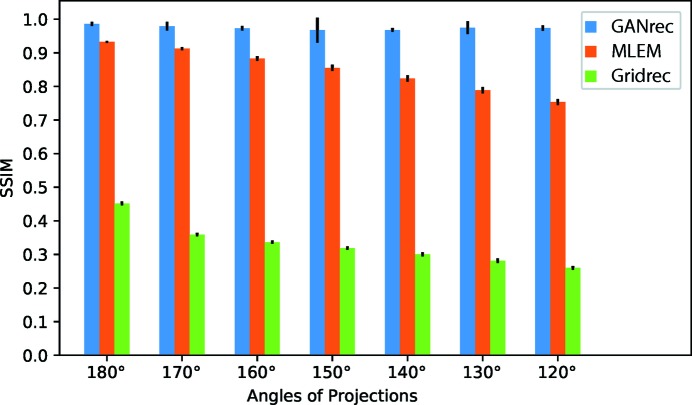
The mean SSIM (MSSIM) of the reconstructions compared with the original object. These MSSIM values were calculated from 128 slices of the 3D object. We compared the MSSIM for GANrec, MLEM and Gridrec from full-angle scanning (0–180°) to (0–120°).

We then plotted the mean SSIM (MSSIM) of all 128 slices for the 3D shale sample (Fig. 5 ▸). The GANrec showed a consistent improvement in the reconstruction quality compared with the MLEM and Gridrec algorithms. The Gridrec had a very low MSSIM (0.45 to 0.26) because of the noise outside the object. The MLEM can remove noise outside the object and had less noise on the structure of the object, so a better MSSIM value was obtained (0.93 to 0.75). The MSSIM decreases consistently for both Gridrec and MLEM when the scanning angle is reduced. The GANrec reconstruction always showed better quality results in these tests (MSSIM 0.98 to 0.97). The standard deviation (Std) changes randomly for different runs of the reconstruction. For instance, the 0–150° case showed a lower MSSIM than the 0–120° case and a higher Std (MSSIM 0.967 *versus* 0.973, Std 0.037 *versus* 0.008). The GANrec algorithm did not show an obvious tendency for quality reduction when the scanning angle was reduced from the full angle to 120°. This indicates that the tomographic reconstruction quality is not strictly limited by the scanning angle when it exceeds specific angles. We therefore further examined extreme cases of missing angles, discovering that the GANrec failed for the case of up to 120° (0–60°) missing. However, we do not claim this is the absolute limit, which is highly dependant on the data conditions, such as the complexity of the pattern, the number of projections and the size of the objective image.

### 3.2. A missing-wedge ptycho-tomographic reconstruction   

Further application of the GANrec to the reconstruction of empirical data was then demonstrated on a missing-wedge ptychographic tomography image series. The zeolite sample investigated here constitutes a suitable case study for missing-wedge tomography reconstruction due to its small size and easily identifiable interior macropore features (Kahnt *et al.*, 2019 ▸). Due to placement on a MEMS chip within the *in situ* sample-holder cell, the zeolite was only accessible from a range of ±35°, leading to a significant missing wedge of 110°. Only 51 projections were recorded for the sample, which is regarded as heavy undersampling.

PXCT measurements were performed on the nanoprobe endstation of beamline P06 at the synchrotron light source PETRA III at DESY (Hamburg, Germany) using the ptychographic nanoanalytical microscope (PtyNAMi) (Schroer *et al.*, 2017 ▸; Schroer *et al.*, 2019 ▸). Measurement parameters were detailed in previous work (Fam *et al.*, 2019 ▸) and are summarized here. An incident X-ray beam of 9 keV was focused using Fresnel zone plates (125 µm aperture, 70 nm outer width) to a spot size of 60 nm. The sample was positioned 0.6 mm downstream from the focal point, leading to an effective beam spot size of 2 µm. The sample was measured in a 12 × 12 grid (144 scan points) with a 333 nm step size and an exposure time of 500 ms per point, leading to a scan time of approximately 6 min per projection. Far-field diffraction patterns were recorded using an EIGER X 4M detector (DECTRIS Ltd, Switzerland) positioned 3.470 m downstream of the sample.

The sample was a single zeolite particle with diameter of *ca* 2–4 µm, containing a system of approximately spherical macropores up to *ca* 600 nm diameter, the preparation and characterization of which have been described in the literature (Machoke *et al.*, 2015 ▸). The sample itself was placed on a ‘Wildfire’ MEMS chip (DENSsolutions, Delft, The Netherlands) using focused ion beam (FIB) micromanipulation with a SCIOS Dual-beam FIB (FEI, USA), performed at DESY Nanolab (Hamburg, Germany). The MEMS chip was placed in a sample-holder cell designed for *in situ* ptychography and PXCT measurements, described in previous work (Fam *et al.*, 2019 ▸). Here, the sample-holder cell was operated under ambient gas and temperature conditions, therefore ptychographic measurements were performed *ex situ*. Due to the cell design and steel frame, which permit accurate temperature control and gas-tight operating conditions, PXCT measurements are currently possible with geometric limitations from ±35° (fully assembled cell) to ±65° rotation (partly assembled cell) and a corresponding missing wedge of 110 to 50°. Here, ptychographic projections were obtained as described above across an angular range of ±35° in 1.4° steps, leading to a total of 51 projections.

**Figure 6:**
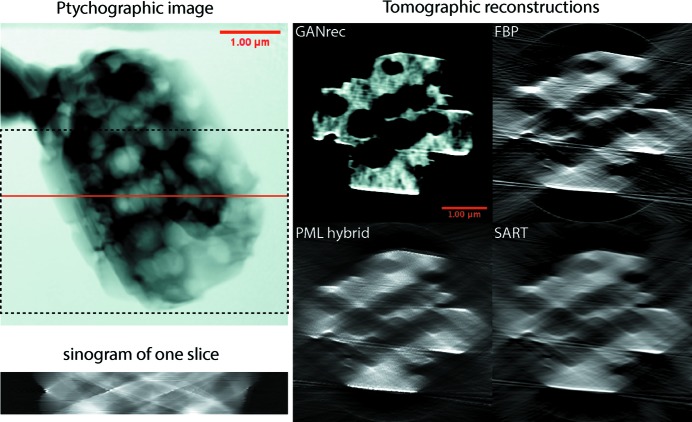
Comparison of tomographic reconstructions for one slice of the zeolite particle. The image in the top left is the ptychographic reconstruction of one angle. The solid red line marks the slice used for the bottom sinogram. The dashed black line marks the region of the tomographic reconstruction in Fig. 7 ▸. The four images on the right are the tomographic reconstructions for the sinogram on the left. GANrec reconstructed the image without significant artefacts and deformation compared with the results from FBP, PML hybrid and SART.

**Figure 7:**
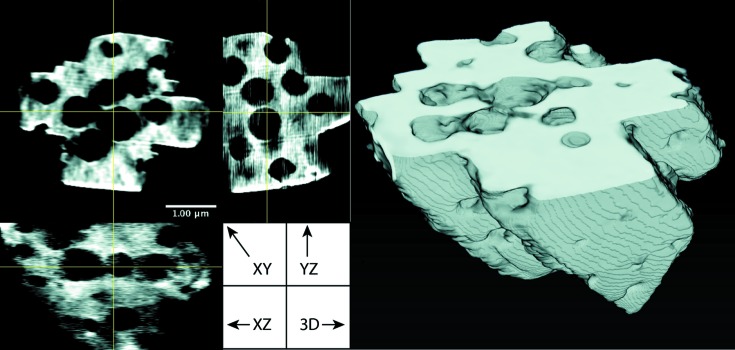
A 3D reconstruction of the zeolite particle using GANrec.

The extreme data-acquisition conditions result in the failure of traditional reconstruction algorithms to produce an acceptable result (Fig. 6 ▸). The GANrec successfully reconstructed the overall structure of the particle, allowing the macroporous interior to be segmented by simple thresholding. The resulting 3D reconstructions from the GANrec algorithm are shown in Fig. 7 ▸. The interior macroporous structure of the particle is a close match to electron microscopy images recorded during preparation and to previous results of electron nanotomography and PXCT with a full 180° rotational range (Machoke *et al.*, 2015 ▸; Weissenberger *et al.*, 2019 ▸).

## 4. Discussion and future work   

As shown in the above results, the GANrec algorithm can learn from the physics model, the Radon transform, to predict the inversion with very good accuracy. Model-based learning is more flexible for applying deep learning to physics problems, especially during data processing of experimental measurements. The preparation of the training data was always a big obstacle for these applications. Our current development of the GANrec does not require any training data but has the advantage of the deep learning method. The complexity of the networks offers a higher chance of finding solutions for ill-posed inverse problems than traditional methods. The results of both simulations and experiments showed that the GANrec can reconstruct extreme missing-wedge data with high quality, which traditional algorithms cannot do effectively.

GANrec is an iterative reconstruction method. It requires much more computational power than traditional algorithms. We tested it on a node of a GPU cluster with four NVIDIA Tesla P100 processors. We distributed the computing of the generator and discriminator on two GPUs. If the reconstruction object is continuous along the rotation axis, we can reconstruct the first slice with several iterations (∼1000–2000 ▸) and use the weights of that reconstruction as the initial weights for the remaining slices. Once the weights of the networks are initialized properly, a small number of iterations (<100) are enough for a good-quality reconstruction. In our computing setup, GANrec took ∼0.07 s for each iteration with an image size of 256 × 256 pixels.

GANrec has the advantage of excellent accuracy in the specific situation of extremely undersampled data. However, its speed is not competitive compared with traditional algorithms. It can be considered as an alternative method in cases where a complete data set cannot be measured, such as the presented limited-angle tomography, and traditional reconstruction algorithms suffer greatly from the missing data. As the hardware and software for deep neural networks are improving rapidly, GANrec will have broader applications in the future. The code of the GANrec will be made available in the *xlearn* toolbox on github in the near future (https://github.com/tomography/xlearn.git).

The success of GANrec in producing reasonable 3D reconstructions from missing-wedge data synergizes strongly with the *in situ* sample cells developed previously for use on beamline P06. To perform ptychography or PXCT under controlled gas and temperature conditions, a certain amount of physical infrastructure will always be necessary, introducing geometric limitations. Providing methods to mitigate these limitations, such as the GANrec, will help to enable successful *in situ* PXCT studies, which are currently rare in the literature.

The framework we have developed for the GANrec can be easily adapted to other inverse problems. One only needs to replace the Radon transform of equations (2) and (3) with the corresponding forward model. For instance, we replaced the Radon transform with Fresnel diffraction propagation to build the phaseGAN, which works with good accuracy for phase retrieval in near-field X-ray imaging. Based on this idea, we are further developing a platform that solves ill-posed inverse problems of X-ray imaging.

## 5. Conclusion   

We developed and implemented the GANrec algorithm for tomographic reconstruction, and validated it using synthetic and real measurement data. This development shows that the GAN is capable of learning the mapping of image transformation directly from the physics model, rather than from the training data sets of the traditional supervised learning approach. Evaluations with synthetic data showed significant improvements in the reconstruction accuracy using the GANrec for missing-wedge tomography. Artefact-free reconstruction can be obtained for missing wedges up to 60° with the GANrec. The algorithm was further applied to an experimentally acquired missing-wedge ptychography image series of a macroporous zeolite, placed within an *in situ* sample holder. A good-quality reconstruction was made by the GANrec even though the tomographic data only had 51 projections recorded over an arc of 70°. Our framework of the GANrec is also suitable for other ill-posed inverse problems if the forward physics model is available.
